# Clinical Implications of Circulating Tumor Cells of Breast Cancer Patients: Role of Epithelial–Mesenchymal Plasticity

**DOI:** 10.3389/fonc.2015.00042

**Published:** 2015-02-26

**Authors:** Linda M. McInnes, Natalie Jacobson, Andrew Redfern, Anthony Dowling, Erik W. Thompson, Christobel M. Saunders

**Affiliations:** ^1^School of Surgery, The University of Western Australia, Perth, WA, Australia; ^2^Medical Oncology, Royal Perth Hospital, Perth, WA, Australia; ^3^Department of Medical Oncology, St Vincent’s Hospital Melbourne, Melbourne, VIC, Australia; ^4^Institute of Health and Biomedical Innovation, School of Biomedical Sciences, Queensland University of Technology, Brisbane, QLD, Australia; ^5^St. Vincent’s Institute, Melbourne, VIC, Australia; ^6^Department of Surgery, St Vincent’s Hospital, University of Melbourne, Melbourne, VIC, Australia

**Keywords:** circulating tumor cells, epithelial–mesenchymal transition, breast cancer, clinical application, metastasis

## Abstract

There is increasing interest in circulating tumor cells (CTCs) due to their purported role in breast cancer metastasis, and their potential as a “liquid biopsy” tool in breast cancer diagnosis and management. There are, however, questions with regards to the reliability and consistency of CTC detection and to the relationship between CTCs and prognosis, which is limiting their clinical utility. There is increasing acceptance that the ability of CTCs to alter from an epithelial to mesenchymal phenotype plays an important role in determining the metastatic potential of these cells. This review examines the phenotypic and genetic variation, which has been reported within CTC populations. Importantly, we discuss how the detection and characterization of CTCs provides additional and often differing information from that obtained from the primary tumor, and how this may be utilized in determining prognosis and treatment options. It has been shown for example that hormone receptor status often differs between the primary tumor and CTCs, which may help to explain failure of endocrine treatment. We examine how CTC status may introduce alternative treatment options and also how they may be used to monitor treatment. Finally, we discuss the most interesting current clinical trials involving CTC analysis and note further research that is required before the breast cancer “liquid biopsy” can be realized.

## Introduction

Breast cancer is the most common cause of cancer death among women (1). Prognosis for most patients with early breast cancer (EBC) is generally very good, however, a significant proportion (20–30%) of chemotherapy-treated EBC patients relapse with metastatic disease (2). How to identify those breast cancer patients who will relapse in the future and develop metastatic disease remains elusive. Metastatic disease is initiated by circulating tumor cells (CTCs) that originate from the primary tumor and spread the cancer in the body via the blood circulatory system. These CTCs may migrate to, and remain dormant in, sites such as bone marrow as disseminated tumor cells (DTCs). After variable latency periods, DTCs may develop into overt metastases and although this is not seen in all patients, it is seen more frequently in breast cancer patients with persistent DTCs (3). Although considerable research has been conducted to characterize these cells and their role in dissemination, dormancy, and formation of metastasis, many questions remain. For example, why are CTCs not detectable in some patients with metastases, and why is it that some patients with detectable CTCs never develop metastases?

In a rat model, human mammary tumors have been shown to shed 3.2–4.1 × 10^6^ cells per day per gram of tissue (4), most of which (~85%) are destroyed within minutes in the circulation (5) by anoikis, a form of apoptosis driven by loss of cell–cell interactions (6). However, some cells are resistant to anoikis (5). In a mouse model, approximately 2.5% of CTCs formed micrometastases (most of which subsequently disappeared over time) and 0.01% of CTCs progressed to form macrometastases (7). Metastatic potential is not only influenced by CTCs resistance to anoikis, but also the ability of CTCs to change their cellular phenotype from epithelial to mesenchymal – termed epithelial–mesenchymal plasticity (8).

Detection of either CTCs or DTCs is commonly associated with an increased risk of metastases and accompanying poor prognosis (9, 10). Researchers have, however, reported considerable variation in CTC detection rates and correlation with prognosis, even in patients with substantial metastatic disease (11). To date, this has prevented the use of CTCs as a routine prognostic clinical tool (12). We focus our review on CTCs, their role in breast cancer progression, and how CTC molecular variation and epithelial–mesenchymal transition (EMT) may explain discrepancies in CTC detection, therapy response, and relationship to prognosis.

## CTC Characteristics

Circulating tumor cells are extremely rare, with a frequency of typically 1 per 10^6–7^ leukocytes (13). Defining characteristics to delineate CTCs from other blood cells is difficult due to the substantial pleomorphism CTCs exhibit (14). Breast cancer CTCs have a mean diameter of 13.1 μm (15), which is only slightly larger than blood leukocytes measured at 10 μm (16). Accepted CTC characteristics include presence of a nucleus, visible cytoplasm, and the expression of cytokeratin and absence of CD45 expression (17).

Clusters of CTCs, also called tumor microemboli, are found in some patients, comprising 4% of CTCs analyzed in one study (14) and have been demonstrated to form prior to entering the circulation, and to be precursors with more malignant potential than their unicellular counterparts (18). Cluster presence, particularly if sustained through treatment, correlates more strongly with poor prognosis than single CTCs do in metastatic breast cancer (MBC) patients (18).

## Epithelial–Mesenchymal Transition

Most breast cancers are of epithelial origin (19). Epithelial cells collectively maintain organized tissue architecture through distinct contact between cells facilitated by E-cadherin, a homotypic transmembrane cell–cell adhesion protein (20, 21). A critical step in tumor invasion and metastasis is the phenotypical change known as EMT, normally a highly regulated process involved in embryogenesis and wound healing, and implicated in several disease states including malignancy and fibrosis (22). Physiologically, activation of a range of highly controlled signaling molecules triggers EMT in response to specific stimuli (23). However in cancer cells, activation of this process is dysregulated (22). During EMT, adhesion molecule expression is altered and cells take on mesenchymal characteristics, becoming more elongated, flexible, mobile, and thereby potentially invasive (19). This phenotype also mediates increased resistance to common anti-cancer therapies including taxanes and anthracyclines (24) and is elevated in breast cancer tissues remaining after neoadjuvant therapies (25). Tumor cells surviving in the hostile environment of the blood have undergone demonstrated EMT changes (26, 27), which are considered crucial to the metastatic process (28) and to resistance to anoikis (29). EMT is, for instance, most evident in “triple-negative” tumors [those without estrogen receptors (ER), progesterone receptors (PR), and human epidermal growth factor receptor 2 (HER2)] and HER2 positive (HER2+) tumors, and least frequent in ER positive (ER+) tumors, particularly lobular cancers, mirroring the metastatic potential of these tumor types (27, 30).

Whilst EMT/mesenchymal markers have been demonstrated on CTCs, breast cancer metastases in liver, lung, and brain often express higher levels of E-cadherin and hence are often “more epithelial” than the primary tumor, indicating a reversal of the EMT process (31), termed mesenchymal to epithelial transition (MET). Evidence for the importance of this reverse transition and its role in metastasis is growing rapidly (32–36).

## Sub-populations of CTCs

Circulating tumor cells can exist in intermediate states – sub-populations expressing both epithelial and mesenchymal markers to varying degrees (27, 37–40). This is likely to be considerably more common than complete polarization to either state (41). Sub-populations of tumor cells at any point may also acquire cancer “stem-cell” (CSC) attributes such as quiescence, self-renewal, asymmetric division, drug resistance (38, 42), and resistance to radiation (43), facilitating survival in the circulation and resultant metastasis. Breast CSCs are most commonly identified with a CD44+/CD24− phenotype (44) or by the expression of aldehyde dehydrogenase 1 (ALDH1) (45). These CSC markers have been identified in breast cancer CTCs populations by a number of researchers (46–49).

## CTC Isolation Methodologies

Current CTC detection methods rely on CTC physical properties (e.g., size, density, electric charge, and cell deformability) or on the retained expression of surface proteins (predominantly epithelial) or messenger RNA. Although there are currently numerous CTC detection methodologies [comprehensive reviews (13, 50–53), the CellSearch system (Veridex, USA)], an immunomagnetic bead capture system based on epithelial cell adhesion molecule (EpCAM), followed by immunofluorescence analysis predominates, as it is the only current method to achieve Federal Drug Administration approval. As malignant cell transcriptional profiles vary, especially during processes such as EMT and CSC formation, expression of identifying proteins may be lost in CTCs as well as being present in non-CTCs, reducing sensitivity and specificity. Barriere et al. (54) reviewed studies exploring CTC isolation, noting their propensity to co-express epithelial, mesenchymal, and CSC markers, and recommended development of a combined isolation method targeting all three phenotypes to avoid missing clinically relevant CTC sub-populations.

## CTCs as Prognostic Tools

Both CTCs and DTCs have been detected in breast cancer patients with disease states ranging from ductal carcinoma *in situ* to MBC (55–60), and their detection is generally associated with a poor prognosis. Although CTCs are not seen in all MBC, this may be due to the inability of current methods to detect EMT sub-populations (54, 61, 62). Extensive studies in MBC show that CTCs associate with disease progression (57, 63, 64) with a meta-analysis by Zhang et al. (10) confirming CTC presence to be an independent prognostic factor for overall survival (OS) in MBC (HR = 2.33, *p* < 0.005).

Links with CTC presence and prognosis in EBC are also suggested (65, 66). Confirming this, a defining meta-analysis by Zhang et al. (10) showed the presence of CTCs to be an independent prognostic factor for OS in EBC (HR = 2.78, *p* < 0.005).

The association of CTCs and prognosis in EBC appears independent of tumor grade, histological type, degree of nodal involvement, lymphovascular invasion, or Ki-67 (proliferation marker) status (67, 68). Mixed results have been seen when considering receptor-defined breast cancer subtypes. Detection of CTCs is prognostic in EBC patients with “triple-negative” tumors or ER negative (ER−) PR negative (PR−) HER2+ primary tumors, but not in patients with ER+ tumors (69). In contrast, Giordano et al. (70) found CTCs to be prognostic in all MBC disease subtypes except HER2+ tumors, whilst Liu et al. (71) found the contrary.

The prognostic importance of CTCs over long-term follow-up has not been established. CTCs have been detected in patients in prolonged remission with 36% of patients in one study having detectable CTCs 8–22 years out from treatment of EBC, despite no clinical evidence of disease (72). What proportion of these patients will go on to develop metastatic disease is not known, nor have the beneficial effects of CTC-guided intervention been established (see Monitoring Treatment – Clinical Utility section below).

## Receptor Discordance

Amplification of the HER2/*neu* gene and subsequent HER2 protein overexpression is associated with significantly decreased disease-free survival (DFS) and OS in the absence of HER2-targeted therapy (73, 74). Similarly, patients with HER2+ CTCs have been reported to have worse progression-free survival (PFS) and OS in comparison with patients with HER2− CTCs or any detectable CTCs (75–77). Heterogeneous amplification of HER2 is, however, known to occur within tumors and this serves to confound HER2 diagnostics and studies of receptor discordance (78). Receptor discordance refers to differences in receptors of primary tumor and metastatic tumors or CTCs. Discordance in HER2 status between primary tumor and CTCs reports are variable, in the order of 15–35% in MBC (75, 79, 80). HER2 discordance has also been reported in EBC patients. Wulfing et al. (77) found that, in EBC patients with detectable CTCs, 12 of 24 (50%) patients with HER2− primary tumors had HER2+ CTCs, and 1 of 3 (33%) patients with HER2+ primary tumors had HER2− CTCs. A few studies have shown that trastuzumab treatment is effective in eliminating HER2+ CTCs, including from patients with HER2− primary tumors and significantly reduced the risk of relapse and prolonged the DFS (81, 82).

Clinical trials are underway testing the utility of CTCs as a therapy decision-making tool in such cases of observed discrepancy in HER2 positivity between the primary tumor and CTCs. The DETECT III trial is randomizing HER2− MBC patients with HER2+ CTCs to standard therapy with or without lapatinib, a HER2-targeted therapy. The TREAT-CTC trial is randomizing HER2− EBC patients with detectable HER2+ CTCs post-neoadjuvant therapy (NT) and surgery to either standard care or additional trastuzumab. These studies may provide a foundation for the use of CTCs in standard clinical practice to identify patients who may benefit from the addition of HER2−directed therapy.

Discordance between the ER and/or PR status of primary and metastatic tumors has long been observed (83–85). Given the role of CTC in progression to metastases, it is not surprising that the hormone receptor status of CTCs may also differ from that of the primary tumor. Interestingly, this discordance appears much greater than that seen between primary and metastatic tissue, implying that receptors may be lost then regained once overt metastases form. Aktas et al. (86) found that discordance rates between primary tumor and CTCs for ER and PR in MBC patients were 59% and 55%, respectively, with most CTCs being ER− and PR− (84% and 92%). Fehm et al. found discordance rates between primary tumors and CTCs in EBC for ER and PR to be 71% and 75%, respectively (87) and HER2 discordance rates in MBC patients to be 36% (76). Although this suggests that hormone receptor loss may often be a transient phenomenon connected with the CTC state, this “sanctuary phenotype” could still contribute to endocrine therapy failure.

As with HER2 discordance; there are also implications for treatment of ER+ CTCs where the primary tumor is ER−. It remains to be seen if estrogen-targeted treatments in ER− primary tumors with ER+ CTCs have a therapeutic effect.

## Monitoring Treatment – Clinical Utility

Another important clinical area uses CTCs as an early marker of disease progression or treatment failure – potentially giving an indication of a need for change of therapy before conventional imaging and/or tumor markers demonstrate progression. The lack of a reliable method to monitor the effects of adjuvant systemic therapy in particular is a significant area of need. Multiple studies have shown that in EBC, locally advanced breast cancer, and MBC, detection of CTCs after the completion of treatment is a strong prognostic marker (58, 64, 88–90).

Circulating tumor cell changes in MBC response to treatment can yield important prognostic information. For instance, MBC patients in whom initially high CTC counts reduced to low levels after initial therapy, had identical prognosis to CTC-negative patients (64). Correlations between the changes in CTC numbers and an objective response to therapy as assessed by serial imaging were reported by a study conducted by Nakamura et al. (91). Pachmann et al. (92) showed that patients who had higher CTC numbers that declined following treatment had a better prognosis than those whose CTC count did not change. Utilizing this paradigm, the SWOG SO500 trial evaluated switching therapy in MBC patients after one treatment cycle if certain CTC fall thresholds were not met. This trial did not demonstrate that an early switch improved DFS or OS, but presence of CTCs was an adverse prognostic factor (93). It has been suggested that the reason for the failure of this trial to observe a benefit to switching treatment on the basis of CTC levels is due to the fact that breast cancers with acquired chemo-resistance to one agent rarely exhibit high sensitivity to a randomly chosen alternative chemotherapeutic agent (94).

There are a number of ongoing clinical trials examining the utility of CTCs in breast cancer treatment. Details of some of the interventional studies employing CTC assessments, which are currently being run, are shown in Table 1. Results are eagerly awaited from the CirCe01 trial, which has similar design to the SWOG SO500 trial but evaluates CTCs serially after each cycle, with patients in the intervention arm changing therapy if CTC counts are adverse (see Table 1). Currently, we do not have clinical trial results supporting the use of CTCs to guide clinical decisions. Bardia et al. (94) highlighted the need for future clinical trials to utilize CTC isolation methodologies that are able to isolate CTCs which have undergone EMT, and to genotype CTCs in order to evaluate therapeutic response and guide therapeutic choices.

**Table 1:** **A selection of current ongoing clinical trials examining the clinical utility of circulating tumor cells in breast cancer treatment**.

Trial name (ClinicalTrial.gov registry number)	Rationale	Patient group	Methodology	Estimated accrual completion date
CTC-EMT (NCT02025413)	Evaluating a novel mesenchymal-marker-based ferrofluid (N-cadherin or O-cadherin based) CTC capture method.	Metastatic prostate or MBC patients	Non-randomized study to evaluate novel CTC capture method.	December 2014
STIC CTC METABREAST (NCT01710605)	Evaluating the medico-economic value CTCs provide in deciding on first-line therapy.	HR+, HER2−MBC patients	Randomized study where patients with ≥5 CTC/7.5 ml blood receive chemotherapy and those with <5 CTC/7.5 ml receive endocrine therapy.	February 2015
COMETI P2 (NCT01701050)	Evaluating the algorithm CTC-Endocrine Therapy Index (CTC-ETI) for the identification of patients that will progress.	ER+, HER2−MBC patients	ER, B-cell lymphoma-2 (BCL2), HER2, and Ki-67 markers assessed on isolated CTCs and CTC-ETI determined.	December 2015
Treat-CTC (NCT01548677)	EBC, HER2−primary tumor patients with no overt metastasis having completed (neo) adjuvant chemotherapy and surgery.	HER2−, CTC+ EBC patients	Patients randomized in 1:1 ratio to either the trastuzumab arm or the observation arm.	April 2017
CTC-CEC-AND (NCT02220556)	Evaluation of different analysis methods for CTCs, CECs, and circulating tumor DNA in patient followed for a tumoral pathology.	Patients with solid tumors	Fifteen cohorts. Each cohort will test one analysis method and/or tumoral type. Up to 50 patients in each cohort.	December 2015
CirCe01 (NCT01349842)	Evaluation of the use of CTCs to guide chemotherapy from the third-line of chemotherapy for MBC.	Advanced MBC patients	Patients with ≥5 CTCs/7.5 ml before third-line of chemotherapy randomized between CTC-driven and standard treatment.	January 2018
DETECT III (NCT01619111)	A multicenter, phase III study to compare standard therapy ± Lapatinib in HER2**−**MBC patients with HER2+ CTCs.	HER2−MBC patients with HER2+ CTCs	Patients randomized between standard therapy ± Lapatinib. Patients with bone metastases treated with denosumab.	March 2018

Circulating tumor cells have been studied with respect to their potential to inform patient therapy. Pierga et al. (95) found a significant correlation between CTC detection before NT and reduced DFS, but no correlation between the persistence of CTCs post-NT and tumor response. Boutrus et al. (96) also found that CTC presence predicted local and distant relapse, but did not correlate with primary tumor volume reduction. Similarly, Riethdorf et al. (68) showed that CTC detection before NT did not correlate with tumor response to treatment, nor did CTC changes necessarily mirror treatment response. This suggests differential responses to treatment between the primary tumor and CTCs.

A large neoadjuvant chemotherapy study conducted by Rack et al. (60) in EBC patients found separate prognostic importance for the presence of CTCs pre- and post-treatment. Interestingly, the initially CTC-negative patients who subsequently developed CTCs fared better than initially CTC positive patients whose CTCs disappeared post-treatment, suggesting CTC clearance does not predict chemotherapy benefit (60).

To date, few studies have examined drug resistance in CTCs. Gradilone et al. (97) evaluated CTC of 42 MBC patients for expression of multi-drug resistance-related proteins (MRPs) and/or ALDH1, a putative tumor-initiating cell/CSC marker that correlates with resistance to some chemotherapeutics. The expression of MRPs on CTCs was found to be predictive of poor response to chemotherapy and significantly correlated with reduced PFS in MBC patients. Patients with CTCs expressing two or more MRPs had shorter PFS than those with CTCs expressing zero or one MRP (7.1 versus 16.4 months; *p* = 0.004). Furthermore, the expression of ALDH1 on CTCs was correlated with MRPs (and the number of MRPs expressed (*p* = 0.000) as well as an increased resistance to chemotherapy. Gazzaniga et al. (98) screened 105 cancer patients (of which 14 had breast cancer) for CTCs and then evaluated the MRP profile of the CTCs, postulating that this could delineate chemotherapy responders from non-responders. Patients were classified as chemotherapy “resistant” or “sensitive” on the basis of their CTC MRP profile, together with the chemotherapy regime the patient had received. This study found that the MRP profiles of patients’ CTCs to be highly predictive of response to chemotherapy, independent of tumor type and stage of disease.

## Conclusion

The presence of CTCs is a powerful independent prognostic factor in both MBC and EBC. However, we increasingly understand that CTCs are heterogeneous, even within an individual patient at different times in the disease trajectory (27, 99). This includes in the receptors that they express, in relation to either the primary tumor or any metastatic disease, as well as in their variable expression of epithelial and mesenchymal markers.

Although CTC count changes are predictive of outcome in MBC, this is largely a disease where serial agents are delivered with palliative intent. Hence, early tailoring of therapies may not greatly impact on outcome. To date, clinical trials have shown that absolute CTC count alterations or CTC persistence do not predict strongly for neoadjuvant response, or improved adjuvant or metastatic outcomes, and hence currently do not provide clinically useful information to drive changes in therapies. With the maturation of the current clinical trials and further developments in the molecular characterization of CTCs, this information will hopefully become available.

Further work is needed, looking at CTC sub-populations including the presence and importance of EMT and CSC populations, and their alteration with treatment. There may be potential for targeting of otherwise treatment-resistant CTCs through novel targets on such populations. Additionally, the appearance of established therapeutic targets such as HER2 and ER on CTCs not present on the primary tumor is of considerable clinical importance, and the results of ongoing HER2-targeting trials are awaited with interest.

The promise of a “liquid biopsy” to diagnose, characterize, monitor, and influence treatment of cancer is still some way off. However profiling the presence and molecular characteristics of CTCs is very likely to provide important predictive and prognostic information in both early and MBC, and may prove useful in assessing response to treatment and as an early warning system for disease recurrence.

## Conflict of Interest Statement

The authors declare that the research was conducted in the absence of any commercial or financial relationships that could be construed as a potential conflict of interest.
